# Chromosome-level genome assembly of the endangered plant *Tetraena mongolica*

**DOI:** 10.1093/dnares/dsad004

**Published:** 2023-03-31

**Authors:** Bingru Liu, Xiaoyu Zhao, Ziyin Wang, Huili Liu, Xueshuang Huang, Peng Yang

**Affiliations:** Key Laboratory of Ecological Protection of Agro-pastoral Ecotones in the Yellow River Basin, National Ethnic Affairs Commission of China, School of Biological Science and Engineering, North Minzu University, Yinchuan 750021, China; Key Laboratory of Ecological Protection of Agro-pastoral Ecotones in the Yellow River Basin, National Ethnic Affairs Commission of China, School of Biological Science and Engineering, North Minzu University, Yinchuan 750021, China; Key Laboratory of Ecological Protection of Agro-pastoral Ecotones in the Yellow River Basin, National Ethnic Affairs Commission of China, School of Biological Science and Engineering, North Minzu University, Yinchuan 750021, China; Key Laboratory of Ecological Protection of Agro-pastoral Ecotones in the Yellow River Basin, National Ethnic Affairs Commission of China, School of Biological Science and Engineering, North Minzu University, Yinchuan 750021, China; Hunan Provincial Key Laboratory for Synthetic Biology of Traditional Chinese Medicine, School of Pharmaceutical Sciences, Hunan University of Medicine, Huaihua 418000, China; Hunan Provincial Key Laboratory for Synthetic Biology of Traditional Chinese Medicine, School of Pharmaceutical Sciences, Hunan University of Medicine, Huaihua 418000, China

**Keywords:** *Tetraena mongolica*, genome assembly, xerophytic plant, PacBio HiFi

## Abstract

*Tetraena mongolica* is an endangered xerophytic shrub with high ecological value for the restoration of desert vegetation because of its high tolerance to drought and heat stress. Here, we generated a high-quality chromosome-level reference genome of *T. mongolica* by combining PacBio HiFi data and Hi-C sequencing technologies, which was approximately 1.12 Gb (contig N50 of 25.5 Mb) in size and contained 61,888 protein-coding genes; repetitive sequences comprised 44.8% of the genome. This genome of *T. mongolica* is the first published genome sequence of a member of the order Zygophyllales. Genome analysis showed that *T. mongolica* has undergone a recent whole genome duplication event, and a recent burst of long terminal repeat insertions afterward, which may be responsible for its genome size expansion and drought adaptation. We also conducted searches for gene homologues and identified terpene synthase (TPS) gene families and candidate genes involved in triacylglycerol biosynthesis. The *T. mongolica* genome sequence could aid future studies aimed at functional gene identification, germplasm resource management, molecular breeding efforts, as well as evolutionary studies of Fabids and angiosperm taxa.

## 1. Introduction

The desert shrub *Tetraena mongolica* (2*n* = 4*x* = 56) is one of China’s key endangered plants.^1^ Its distribution is restricted to Inner Mongolia and Ningxia in the East Alashan-West Ordos region.^2^ It is widely known as a ‘living fossil’ and is considered by many as the ‘giant panda’ of the plant world. It has evolved various morphological and physiological features to support its long-term growth in harsh environments with severe drought stress, such as its dwarf stature, well-developed root system, fleshy and small leaves, and abundant hairs; these properties make this plant valuable for wind and sand control, soil and water conservation, and the maintenance of desert ecosystem functions.^3^ However, the genomes of only a few xerophytic plants have been sequenced to date (e.g. *Haloxylon ammodendron*, *Ammopiptantbus nanus*, and *Aloe vera*),^4–6^ yet genomic information is critically important for clarifying the evolutionary and genetic mechanisms underlying their drought tolerance.

Since the 1990s, various natural and anthropogenic factors such as global climate change, excessive logging, industrial development, and pest infestations have induced severe damage to the habitat of *T. mongolica*; populations of this species thus require urgent conservation attention.^7^ Most previous studies have focused on examining the structure and diversity of xeric plant communities, the utility of different breeding techniques, chloroplast genomes, pest and disease surveys, the efficacy of different conservation measures, and analysis of the chemical components and rhizosphere microorganisms of *T. mongolica*.^8–12^ However, there is still much to be learned regarding its genome. *Tetraena mongolica* is classified as a basal group of the Fabids clade according to the APG classification system,^13^ and no comparative genomics studies of this family have been conducted to date. The sequencing and assembly of the *T. mongolica* genome will aid future research examining the evolution, genetic diversity, and conservation of *T. mongolica*, as well as germplasm resource management and molecular breeding programs.

Here, we generated a chromosome-level reference genome of *T. mongolica* using PacBio High-Fidelity (HiFi) sequencing and Hi-C scaffolding. This genome of *T. mongolica* is the first published genome sequence of a member of the order Zygophyllales. We then annotated the genome and conducted a comparative genomic analysis. We identified terpene synthase (TPS) gene family members and several triacylglycerol synthesis-related candidate genes within the genome, which provided insights into volatile oil biosynthesis in *T. mongolica*. This valuable genomic resource will aid future studies of the evolution of *T. mongolica* and related xerophytic species as well as studies of the evolutionary history of Fabids and angiosperm taxa.

## 2. Materials and methods

### 2.1. Plant materials and sequencing

**Figure 1.**
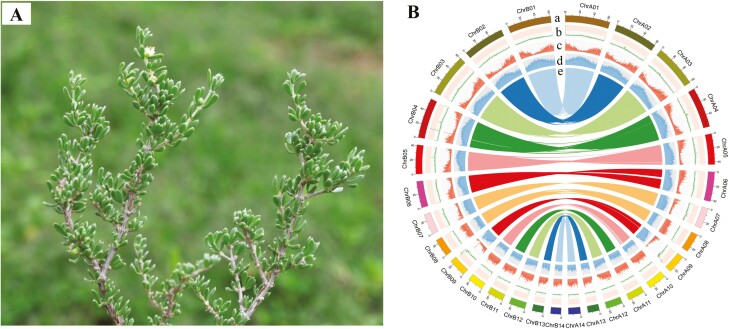
(A) Morphological characteristics of *T. mongolica*. (B) Circos plot of *T. mongolica* genome assembly. The window is 100 kb in size. (a) chromosome karyotypes; (b) GC content; (c) gene density; (d) repeat sequence density; and (e) syntenic blocks.

All samples were obtained from 5-year-old plants of *T. mongolica* in Shizuishan, Ningxia Province, northwestern China (39°22ʹN, 106°45ʹE) (Fig.1A). A DNA secure Plant Kit (Tiangen, China) was used to extract high-quality DNA. For short-read DNA sequencing, paired-end libraries were constructed from 350-bp fragments and sequenced on an Illumina HiSeq 2500 platform (Illumina, San Diego, CA, USA); they were then used for genome survey and assessment. For long-read DNA sequencing, PacBio DNA libraries were built following PacBio’s standard protocol for third-generation sequencing and sequenced on the PacBio Sequel platform to generate circular consensus sequences (CCS) for contig assembly. Hi-C libraries were sequenced on the Illumina HiSeq X Ten System to generate 150-bp paired-end reads, which were used to anchor the scaffolds to the chromosomes. Briefly, DNA isolated from fresh leaves was digested with *Hind*III overnight. To generate chimeric junctions, sticky ends were biotinylated and proximity-ligated; they were then enriched and physically sheared to a size of 300–700 bp. Chimeric fragments representing the cross-linked long-distance physical connections were then used to create paired-end sequencing libraries.

Four tissues (root, stem, leaf, and flower) were used for transcriptome sequencing. Equal concentrations of RNA from different tissues were mixed, and the samples were then sequenced using the Pacbio Sequel II platform. Isoseq3 v3.4.0 (https://github.com/PacificBiosciences/IsoSeq) and Pbmm2 v1.7.0 (https://github.com/PacificBiosciences/pbmm2) were used to process the Pacbio Iso-seq data.

### 2.2. Genome assembly and assessment

Prior to assembly, k-mer frequency distribution analysis of Illumina DNA short reads was conducted using GenomeScope v2.0^14^ to estimate the genome size, heterozygosity, and repeat content of the *T. mongolica* genome. CCS v4.2.0 software (https://github.com/PacificBiosciences/ccs) was used to convert the raw PacBio subreads into HiFi data; the parameter min-passes was set to 3. The HiFi data were then *de novo* assembled using Hifiasm v.0.2.0^15^ with default parameters. Prior to chromosome assembly, we first split the scaffold into fragments with an average size of 50 kb. Hi-C reads were uniquely mapped to the contig assemblies using BWA v0.7.12,^16^ and chromosome-level scaffolds were produced using LACHESIS^17^ (ligating adjacent chromatin enables scaffolding *in situ*). The Core Eukaryotic Genes Mapping Approach (CEGMA) v2.5^18^ and Benchmarking Universal Single-Copy Ortholog (BUSCO) v4.0.6^19^ were used to evaluate the reliability of the genome assembly. The coverage of second-generation DNA data and full-length transcriptome were evaluated by mapping the DNA and Iso-seq data to the genome using BWA v0.7.12^16^ and Minimap2 v2.21,^20^ respectively.

### 2.3. Genome annotation

Transposable elements were identified using a combination of homology-based and *de novo* methods. A library of *de novo* repeats was created using RepeatModeler v2.0.1^21^; LTRharvest v.1.5.10^22^; and LTR_FINDER v1.07^23^ were then used to identify full-length long terminal repeat (LTR) retrotransposons (fl-LTR-RTs). LRT_retriever v2.9.0^24^ was used to generate high-quality intact fl-LTR-RTs and a non-redundant LTR library. Species-specific TE libraries were constructed using the above TE sequence libraries and the Repbase v19.06,^25^ REXdb v3.0,^26^ and Dfam v3.2 databases.^27^ Lastly, repetitive elements were grouped and identified using RepeatMasker v4.1.2.^28^

Homology-based prediction, transcriptome data, and *ab initio* gene prediction methods were used to annotate protein-coding genes. Briefly, Augustus v3.1.0^29^ and SNAP v2006-07-28^30^ were used to predict *de novo* gene models. GeMoMa v1.7^31^ software and reference gene models from four species (*Arabidopsis thaliana*, *Medicago truncatula*, *Populus trichocarpa*, and *Vitis vinifera*) were used for homology-based prediction. Genes were predicted based on the Iso-seq transcripts using GeneMarkS-T v5.1.^32^ Gene models generated via these different methods were integrated using EVM software v1.1.1,^33^ and PASA v2.4.1^34^ was used to polish the resulting gene predictions. The longest transcript was retained for each gene model.

Functional annotation, orthology assignment, and domain prediction were conducted with the protein-coding genes by performing BLASTP searches against the following public databases: Non-Redundant Protein Sequence Database (NR), Eggnog,^35^ TrEMBL,^36^ Pfam (http://pfam.xfam.org), Swiss-Prot,^37^ eukaryotic orthologous groups, gene ontology (GO), and Kyoto Encyclopaedia of Genes and Genomes (KEGG).^38^ Predictions of tRNAs were performed using tRNAscan-SE v1.3.1^39^ and eukaryote parameters. Infernal v1.1^40^ in the Rfam database^41^ with default parameters was used to predict miRNA, rRNA, and snRNA genes.

### 2.4. Comparative genomic analysis

*Tetraena mongolica* subgenome A was used to conduct a comparative genomic analysis. Gene families in *T. mongolica* and 11 angiosperm species (e.g. *Averrhoa carambola*,^42^*A. thaliana*,^43^*M. truncatula*,^44^*V. vinifera*,^45^*P. trichocarpa*,^46^*H. ammodendron*,^4^*Tripterygium wilfordii*,^47^*Cercis chinensis*,^48^*A. nanus*,^5^*Oryza sativa*,^49^ and *Amborella trichopoda*^50^) were identified using OrthoFinder v2.5.1.^51^ Multiple sequence alignments of 774 single-copy homologous genes were performed using MAFFT v7.205,^52^ and a maximum-likelihood phylogeny with 1,000 bootstrap replicates was built using IQ-TREE v1.6.11.^53^ The tree was visualized using iTOL (https://itol.embl.de/). Divergence times were estimated using MCMCTREE in the PAML package v4.9i,^54^ and four secondary calibration points were obtained from the TimeTree database (http://www.timetree.org/): *A. thaliana*-*A. trichopoda* (199.1-179.0 Mya), *O. sativa*-*V. vinifera* (174.8-143.0 Mya), *A. nanus*-*M. truncatula* (79.1-50.9 Mya), and *H. ammodendron*-*M. truncatula* (125.0-112.4 Mya). Expansions and contractions of orthologous gene families were identified using CAFÉ v4.2,^55^ and the threshold for statistical significance for identifying expansions and contractions was *P* < 0.05. ClusterProfile v3.14.0 was used to conduct GO and KEGG enrichment analysis with unique *T. mongolica* genes and *T. mongolica* genes that have undergone expansions.

### 2.5. Whole genome duplication events and long terminal repeat insertion time analysis

Whole genome duplication (WGD) events were inferred according to calculations of the synonymous substitution rates (*Ks*) and four-fold synonymous third-codon transversion rates (4DTv). Syntenic blocks and syntenic genes were identified using JCVI,^56^ and dot plots were used to visualize *Ks* values and inter-subgenomic syntenic blocks. WGD v1.1.1 (https://github.com/arzwa/wgd) was used to calculate *Ks* values between syntenic gene pairs. The Perl script calculate_4DTV_correction.pl (https://github.com/JinfengChen/Scripts/) was used to calculate 4DTv values. The R package ggplot2 was used to visualize the distributions of *Ks* and 4DTv values.

LTRharvest v1.5.10^22^ and LTR_FINDER v1.07^23^ were used to identify LTR sequences in the *T. mongolica* genome, and LTR_retriever v2.9.0^24^ was used to integrate and filter the outputs. After the flanking sequences on both sides of the LTR sequence were extracted, MAFFT v7.205^52^ was used to compare these sequences, and the distance between sequences was calculated using the Kimura model in EMBOSS v6.6.0.^57^ The integration times in Mya of intact LTRs were estimated using the following equation: *T* = *K*/2*r*, *r* = 7 × 10^-9^.

### 2.6. Identification of *TPS* and triacylglycerol biosynthesis genes

To identify candidate *TPS* genes, HMM profiles of Terpene_synth (PF01397) and Terpene_synth_C (PF03936) obtained from the Pfam database (http://pfam.xfam.org) were used to conduct searches against *T. mongolica* protein sequences using HMMER^58^ with an *E*-value cut-off of 1e^−^.^5^ Alignments of TPS protein sequences were conducted using MUSCLE, and IQ-TREE was used to build the maximum-likelihood tree with 1,000 bootstrap replicates. *TPS* gene and protein sequences of *A. thaliana* and *O. sativa* were downloaded from the National Center for Biotechnology Information (NCBI) database to classify the *TPS* genes into different subfamilies. EvolView (https://www.bio.tools/evolview) was used to visualize the phylogenetic tree. Conserved motifs were identified using MEME. TBtools software^59^ was used to analyse and visualize the domain composition of TPS proteins and the structure of *TPS* genes. The promoter sequence of 2,000 bp upstream of the start codon of *TmTPSs* was extracted from *T. mongolica* genome. The promoter cis-acting elements were analysed by online website PlantCARE (http://bioinformatics.psb.ugent.be/webtools/plantcare/html/), and the results were visualized by TBtools software.

To identify genes involved in triacylglycerol synthesis pathways, we first obtained protein sequences from the *A. thaliana* genome, including glycerol-3-phosphate O-acyltransferase (*GPAT*), 1-acyl-sn-glycerol-3-phosphate acyltransferase (*LPAT*), lysophospholipid acyltransferase (*LPEAT*), phosphatidate phosphatase (*PP*), wax ester synthase (*WSD*), and diacylglycerol O-acyltransferase (*DGAT*) from the NCBI database. Iterative BLASTP searches with an *E*-value cut-off of 1e^−5^ were conducted to identify their homologues in the *T. mongolica* genome. JCVI^56^ was used to analyse tandem and segmental replication events in *TPS* genes and triacylglycerol biosynthesis genes, and the results of these analyses were visualized using TBtools.^59^

## 3. Results

### 3.1. A high-quality genome assembly

Sequencing of the *T. mongolica* genome yielded a total of 65.3 Gb (~58×) of HiFi long reads, 247.6 Gb (~220×) of Hi-C data, and 81.9 Gb (~72×) of Illumina short reads (Supplementary Table S1). Prior to assembly, a k-mer analysis of the genome survey sequences revealed that *T. mongolica* is a autotetraploid, with a monoploid genome size of ~580.4 Mb and a diploid genome size of ~1,160.8 Mb (Supplementary Fig. S1 and Supplementary Table S2). The genome was estimated to have a high heterozygosity rate (2.93%), which increased the difficulty of *de novo* genome assembly. A *de novo* assembly of the HiFi long-read sequencing data was generated using Hifiasm, and this yielded a draft assembly of 1,124.6 Mb consisting of 835 contigs (contig N50 of 25.5 Mb). Using the Hi-C data, approximately 1,042.8 Mb sequences (92.73% of the entire assembly) were anchored to 28 pseudochromosomes with sizes ranging from 15.5 Mb to 64.2 Mb (Supplementary Table S3). Moreover, synteny analysis showed that 28 pseudochromosomes of *T. mongolica* can be divided into two homologous groups (Fig. 1B), which indicates that *T. mongolica* is an autotetraploid, and this result was also consistent with that of survey analysis. The final diploid chromosome-level genome of *T. mongolica* contained 28 chromosomes and had a total size of 1.12 Gb, a scaffold N50 of 42.4 Mb, and a contig N50 of 25.5 Mb (Fig. 1B and Table 1).

**Table 1.** Major indicators of the *T. mongolica* genome

**Assembly features**	
Total genome size (Mb)	1,124.6
Contig N50 (Mb)	25.5
Contig number	835
Total scaffolds length (Mb)	1,042.8
Scaffold N50 (Mb)	42.4
Pseudochromosomes	28
GC content (%)	33.14%
Gap number	51
Complete BUSCO (%)	94.5%
Complete CEGMA (%)	99.8%
**Annotation features**	
No. of protein-coding genes	61,888
Average gene length (bp)	3,821
Average CDS length (bp)	1,321
Percentage of repeat sequences (%)	44.76
No. of miRNA	91
No. of rRNA	8,670
No. of tRNA	7,905
Complete BUSCO (%)	98.3%

Approximately 99.26% of the Iso-seq data could be mapped back to the genome, indicating the high completeness of the *T. mongolica* genome assembly. BUSCO analysis using the embryophyta_odb10 database revealed that 95.0% and 0.6% of the genes in the *T. mongolica* genome were complete and fragmented, respectively, and CEGMA indicated that 99.78% of the genome was reliably assembled (Supplementary Table S4). The Hi-C interaction heatmap revealed a high frequency of interactions within individual chromosomes, which indicates that the resolution of the Hi-C assembly was high (Supplementary Fig. S2).

### 3.2. Genome annotation

Structure prediction and *de novo* prediction approaches were used to identify repetitive sequences in the *T. mongolica* genome. Repetitive sequences comprised 44.76% of the genome sequences, including LTRs (28.93%), DNA transposons (12.53%), and interspersed nuclear elements (3.22%) (Supplementary Table S5). The *Gypsy* and *Copia* elements accounted for 10.20% and 10.63% of the LTRs, respectively. According to the *de novo*, homology, and transcriptome gene prediction approaches, a total of 61,888 protein-coding genes were annotated in the *T. mongolica* genome (Table 1). Gene information for *T. mongolica* and the other species (*V. vinifera*, *A. thaliana*, *P. trichocarpa*, and *M. truncatula*) revealed an average CDS length of 1,320.8 bp, average gene length of 3,821.5 bp, average number of exons of 5.37, and average number of introns of 4.37 (Supplementary Table S6). Overall, functional annotations were obtained for 59,761 (96.6%) of the protein-coding genes by at least one public database, and KEGG pathways were assigned to 45,318 (73.2%) of these genes (Supplementary Table S7). Various non-coding RNAs in the *T. mongolica* genome were identified, including 8,670 rRNAs, 7,905 tRNAs, 202 snRNAs, and 91 miRNAs. BUSCO analysis using the embryophyta_odb10 database revealed that 98.33% of the conserved genes were present in our annotations (Supplementary Table S4).

### 3.3. Evolutionary and comparative genomic analysis

**Figure 2.**
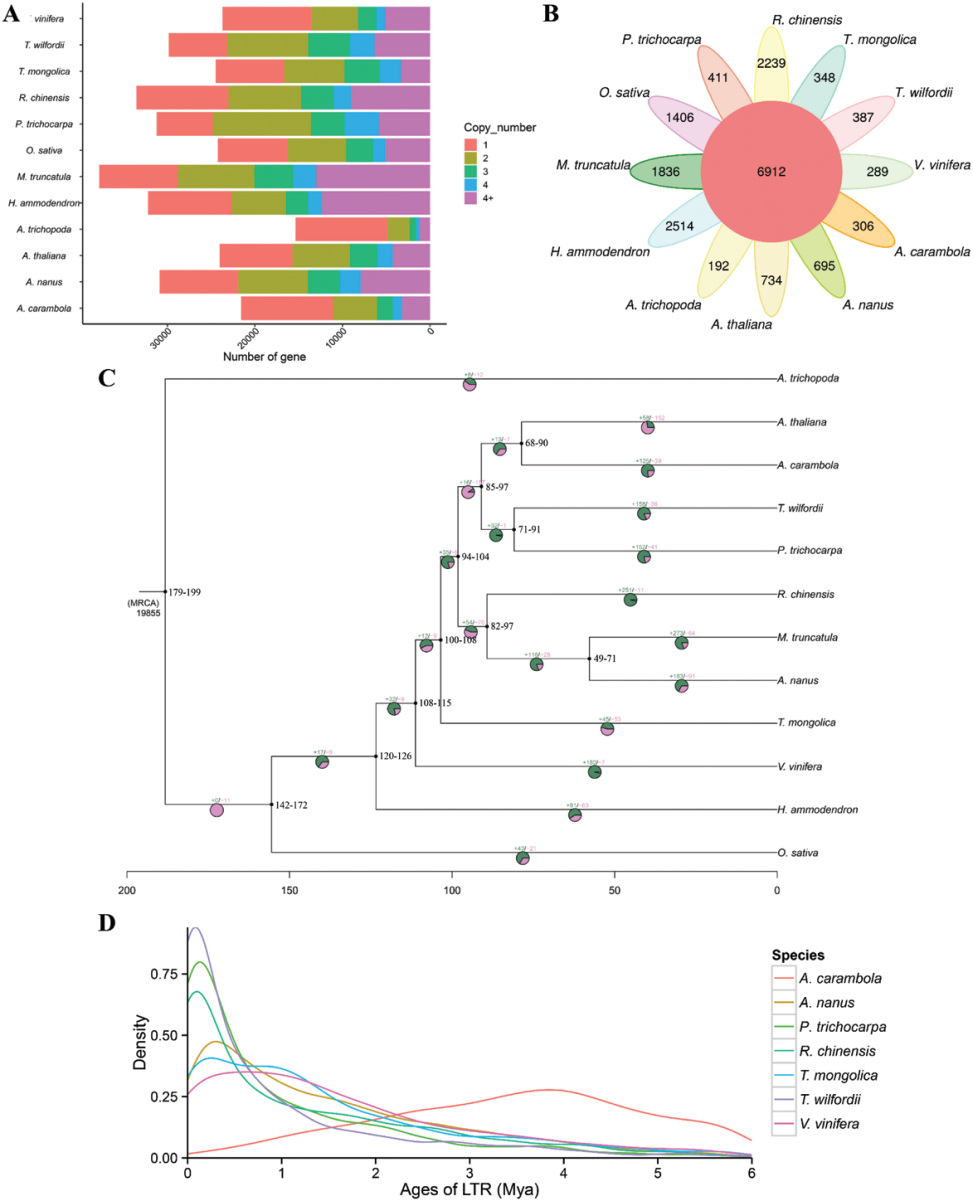
Evolution of the *T. mongolica* genome and gene families. (A) Copy number distribution of the gene families in 12 species. (B) Petal diagram of the gene families in 12 species. The middle circle is the number of gene families shared by all species, and the number of unique gene families is on the side. (C) Phylogenetic tree and gene family expansions/contractions in 12 species. Numbers on nodes represent the inferred divergence times with 95% confidence intervals. (D) Distribution of LTR-RTs insertion time of *T. mongolica* and other six plant species.

We conducted a comparative genomic analysis of the *T. mongolica* genome against 11 other plant species: *A. trichopoda*, *A. thaliana*, *A. carambola*, *T. wilfordii*, *P. trichocarpa*, *C. chinensis*, *M. truncatula*, *A. nanus*, *V. vinifera*, *H. ammodendron*, and *O. sativa*. A total of 24,476 (89.1%) *T. mongolica* genes were clustered into 13,707 gene families, which included 6,912 gene families shared by all species and 348 *T. mongolica*-specific families (Fig. 2a and b and Supplementary Table S8). GO enrichment analysis revealed that the unique *T. mongolica* genes were significantly enriched in the following terms: biological regulation, response to stimulus, transporter activity, and antioxidant activity (Supplementary Fig. S3). KEGG analysis revealed that these unique genes were enriched in several pathways, starch and sucrose metabolism, carbon metabolism, and propanoate metabolism, which indicate that these genes might be involved in drought adaptation (Supplementary Fig. S4). A time-calibrated phylogenetic tree was constructed using 774 single-copy orthologs from 12 species, and the topology of the tree was consistent with our current understanding of the relationships among these taxa (Fig. 2C). *Tetraena mongolica* was allied with another desert plant, *H. ammodendron*, both of which are older species that have been separated for approximately 122 Mya (Fig. 2C). Analysis of gene family evolution revealed significant expansions and contractions of 45 and 53 gene families in *T. mongolica*, respectively (Fig. 2C). KEGG enrichment analysis revealed that the expanded gene families were mainly enriched in glutathione metabolism, starch and sucrose metabolism, and cutin, suberine, and wax biosynthesis (Supplementary Fig. S5), whereas the contracted gene families were enriched in phenylpropanoid biosynthesis, cyanoamino acid metabolism, and isoflavonoid biosynthesis (Supplementary Fig. S6); these genes thus mediate adaptation to the environment, are involved in secondary metabolite synthesis, and likely contribute to phenotypic diversification and even speciation.

**Figure 3.**
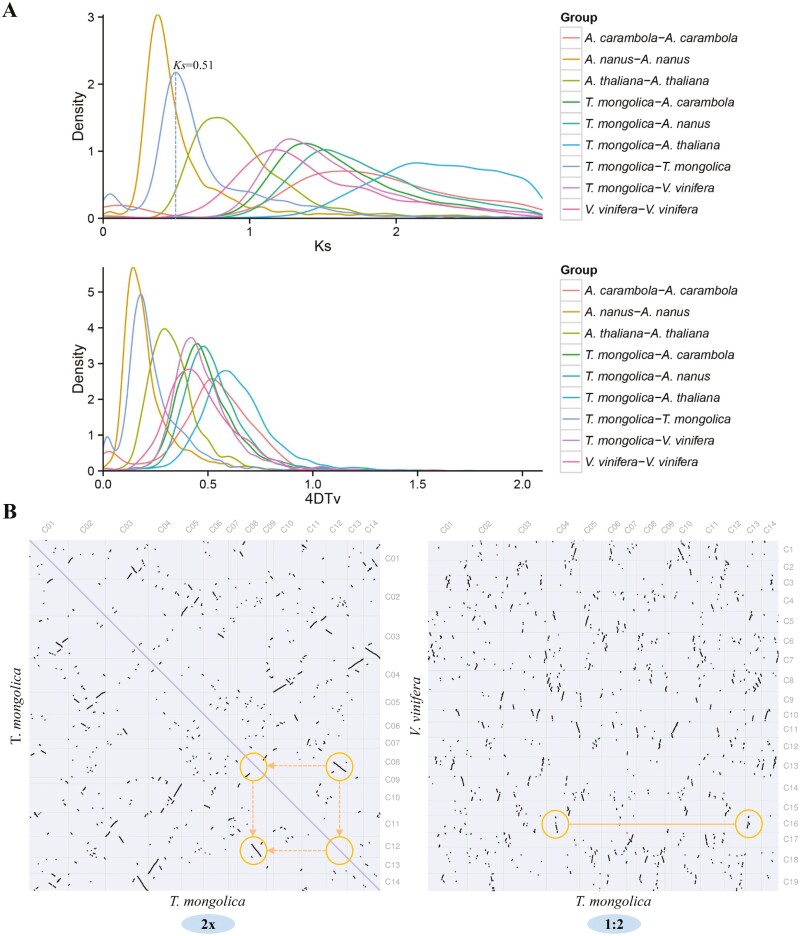
Identification of WGD events in the *T. mongolica* genome. (A) Distribution of *Ks* and 4DTv values between *T. mongolica* and other four plant species. (B) Dot plot of synthenic blocks in *T. mongolica* genome and *T. mongolica* versus *V. vinifera* genome.

We conducted pairwise and self-comparisons between the *T. mongolica* genome and the genomes of four other species (*A. carambola*, *A. nanus*, *A. thaliana*, and *V. vinifera*) and examined the distributions of *Ks* and 4DTv between syntenic genes to identify potential WGD events that might have occurred in the evolutionary history of *T. mongolica* (Fig. 3A). A major peak in the *Ks* distribution of duplicated genes in the *T. mongolica* genome was observed at ~0.51, suggesting that *T. mongolica* has experienced a recent WGD event at approximately 36 Mya. Results of a dot plot analysis of pseudochromosomes also suggested that *T. mongolica* has experienced a recent WGD event (Fig. 3B). We also identified 2,685 intact LTR-RTs in the *T. mongolica* genome. We examined the insertion times of LTRs in the genomes of *T. mongolica*, *A. carambolam*, *A. nanus*, *P. trichocarpa*, *R. chinensis*, *T. wilfordii*, and *V. vinifera* (Fig. 2D). A recent increase in LTRs was observed in the *T. mongolica* genome, and similar median insertion times were observed for the *Copia* and *Gypsy* elements (Supplementary Fig. S7). These findings indicate that the recent WGD event and a recent burst of LTR-RTs in the *T. mongolica* genome have contributed to the amplification of its genome.

### 3.4. Identification of *TPS* genes

**Figure 4.**
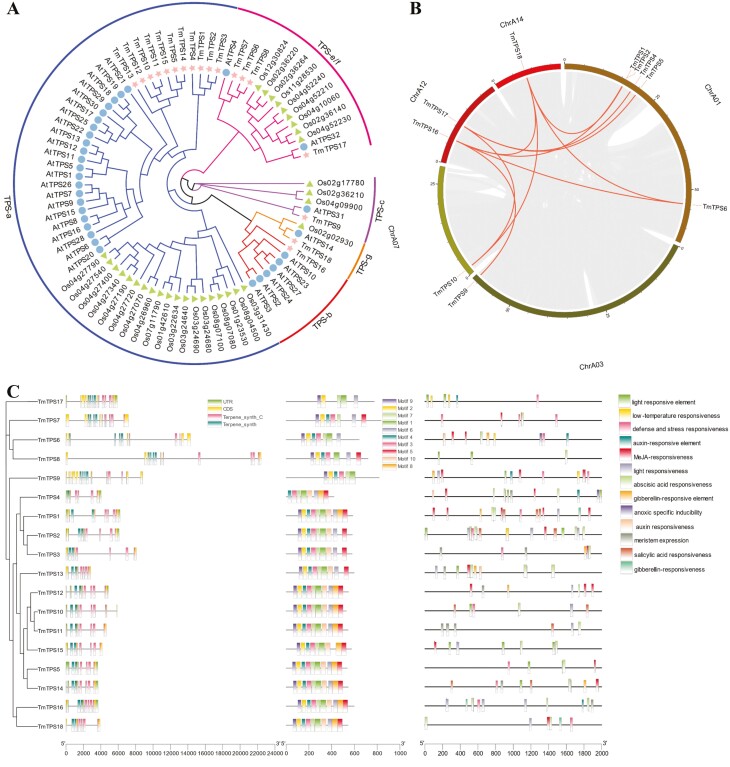
Analysis of TPS gene family in *T. mongolica*. (A) Phylogenetic tree of TPS genes from *T. mongolica* (18 genes), *A. thaliana* (32 genes), and *O. sativa* (32 genes). (B) Chromosome duplication analysis of *TmTPSs* in circos. Highlight lines represent the syntenic gene pairs. (C) Analysis of gene structure, protein structural domain, conserved motif, and *cis*-acting elements of *TmTPSs*.

Terpenoids are one of the main components of the volatile oils in *T. mongolica*, and the *TPS* gene family is responsible for terpenoid biosynthesis and the structural diversity of terpenoids. A total of 18 *TPS* genes were identified in *T. mongolica* subgenome A, including 11 *TPS-a*, one *TPS-b*, one *TPS-c*, four *TPS-e/f*, and one *TPS-g* (Fig. 4A and Supplementary Table S9). More than 60% of the *TPS* genes belonged to *TPS-a*, which encodes proteins involved in the biosynthesis of sesquiterpenoids, indicating that the *TPS-a* gene subfamily has undergone a major expansion. A total of 10 segmentally duplicated gene pairs and 5 tandem duplicated gene pairs were detected among the 18 *TPS* genes, indicating that the expansion of the *TPS* gene family has been driven by these two types of gene duplication events (Fig. 4B and Supplementary Fig. S8). We analysed the structure of *TPS* genes to clarify their functions and evolution (Fig. 4C). With the exception of *TmTPS6* and *TmTPS8*, the exon–intron structure, especially the number of introns and the lengths of the exons, was similar for most genes clustered in the same group. Using MEME software, we identified 10 conserved TmTPS protein motifs, and their lengths ranged from 15 to 41 amino acids (Fig. 4C). Although different motifs were observed in some branches, the motifs of TmTPSs within the same branch were generally similar. Many stress-related and defense-related *cis*-elements were located in the promoter regions of *TPS* genes (Fig. 4C). This information could aid future studies aimed at identifying the functions of *TPS* genes as well as evolutionary analyses of these genes.

### 3.5. Genes involved in triacylglycerol biosynthesis

**Figure 5.**
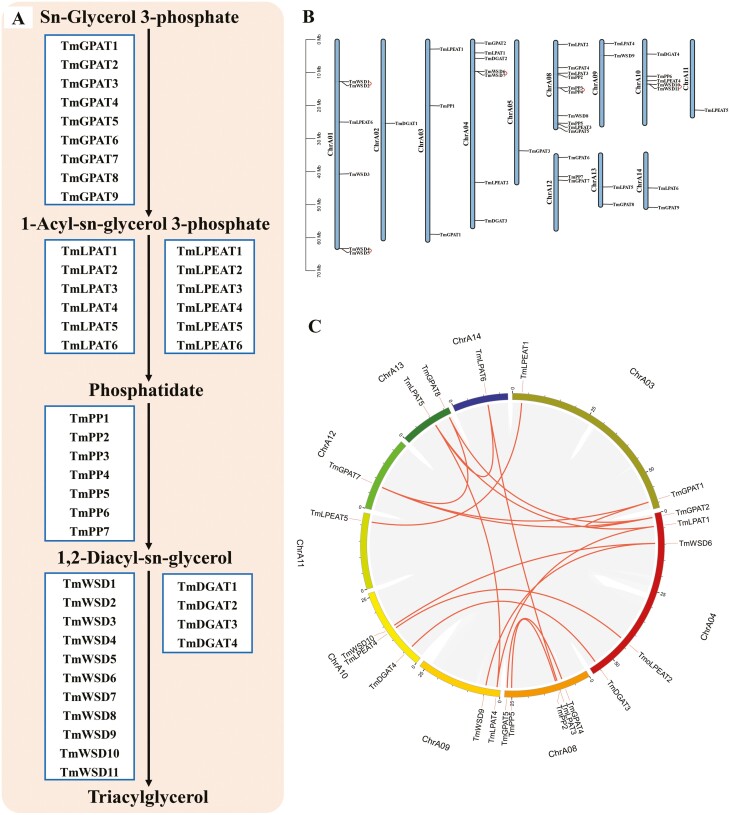
Analysis of genes involved in triacylglycerol biosynthesis in *T. mongolica*. (A) Triacylglycerol biosynthesis pathways *T. mongolica*. (B) Chromosome location and distribution analysis of triacylglycerol biosynthesis genes. Tandem duplicated genes are linked by a highlight line. (C) Chromosome duplication analysis of triacylglycerol biosynthesis genes in circos. Highlight lines represent the syntenic gene pairs.

We identified a total of 43 genes that might be involved in triacylglycerol biosynthesis in the *T. mongolica* genome (Fig. 5A and Supplementary Table S10). Analysis of segmental duplication and tandem duplication events revealed that *GPAT*, *LPAT*, *LPEAT*, *PP*, and *DGAT* have undergone segmental replication events, whereas *WSD* has undergone both segmental and tandem duplication events (Fig. 5B and C). All 43 candidate genes were evenly distributed on the chromosomes. These candidate genes provide important information for future studies aimed at identifying the functions of these genes as well as for the molecular breeding of *T. mongolica*; these genes could also be used to increase the oil content and improve the quality of biodiesel produced from *T. mongolica*.

## 4. Discussion

*Tetraena mongolica* is a relict plant endemic to China that is currently endangered and considered rare; it provides many ecosystem services and is a key species in steppe deserts.^60^ However, studies of the evolution and molecular biology of plants in the Fabids clade have been limited by a lack of high-quality reference genomes of Zygophyllales taxa. We used PacBio HiFi data and Hi-C data to assemble a 1.12 Gb chromosome-level genome for *T. mongolica*, with a contig N50 of 25.5 Mb, which is higher compared with other xerophytic plants, such as *A. nanus* (823.74 Mb, N50 of 2.76 Mb)^5^ and *O. thomaeum* (245 Mb, N50 of 2.4 Mb),^61^ and 95.0% of the genes in the *T. mongolica* genome were complete according to BUSCO analysis. A total of 61,888 genes were annotated in the *T. mongolica* genome, which is more than the number of genes annotated in the genomes of *H. ammodendron* (41,647 genes)^4^ and *C. songorica* (54,383 genes).^62^ This genome is a major milestone in the study of the molecular biology of *T. mongolica*, as it represents the first chromosome-level genome for a species in the order Zygophyllales and the family *Zygophyllaceae*. This genome sequence will aid the germplasm management, molecular breeding, and pharmaceutical and ecological studies of plants in this family.

Phylogenetic analyses indicate that *T. mongolica* is an older species, which is similar to the desert plant *H. ammodendron*.^4^*Ks* and 4DTv analyses both indicate that *T. mongolica* has undergone a recent WGD event, and this has likely increased its genome size and resistance to drought.^63^ The recent increase in LTR-RTs coincides with the aridification of inland Asia in the Late Cenozoic, which might be associated with global cooling or the rapid uplift of the Tibetan Plateau.^64,65^ The total GC content of the *T. mongolica* genome was 33.14%, which was similar to that of other xerophytic plants, such as *H. ammodendron* (35.4%), suggesting that the low GC content in plants might facilitate adaptation to harsh environments limited in nutrients and water.^66^

*Tetraena mongolica* is a highly xerophytic plant rich in volatile oils; however, genes involved in the synthesis of volatile oils have not been identified in *T. mongolica*.^67^ A total of 18 *TPS* genes were identified in this study, 11 of which were *TPS-a* genes involved in sesquiterpenoid synthesis. The classification of the *TPS* family genes in our study was consistent with the classification of terpenoids described in a previous study.^68^ In addition, the number of *TPS* genes in the *T. mongolica* genome is less than that of *Arabidopsis thaliana* and *Oryza sativa*,^69^ suggesting that the volatile oil composition may be mainly triacylglycerols and fatty acids, while the terpenoid content is less. Genes involved in triacylglycerol biosynthesis (*WSD* and *DGAT*) appeared to have undergone a major expansion in the genome, indicating that these genes play a key role in the accumulation of volatile oils.^70^ In addition, segmental duplication and tandem duplication events were the main types of duplication events driving the expansion of these candidate genes in the genome. Additional studies are needed to clarify the functions of these candidate genes and the molecular mechanisms of volatile oil synthesis.

## 5. Conclusion

In sum, the high-quality reference genome described here provides new insights into the genomic evolution and volatile oil synthesis of *T. mongolica*. The genomic data generated in this paper will aid future evolutionary and molecular biological studies of *T. mongolica* and Zygophyllaceae, the conservation of this endangered species, and molecular breeding programs.

## Supplementary Data

Supplementary data are available at *DNARES* online.

**Figure S1.** 21-mer analysis to estimate the *T. mongolica* genome size.

**Figure S2.** Heatmap of the Hi-C interaction density between 28 pseudochromosomes.

**Figure S3.** GO annotations of unique genes in *T. mongolica* genome.

**Figure S4.** The KEGG pathway analysis of unique genes in *T. mongolica* genome.

**Figure S5.** The KEGG pathway analysis of expanded genes in *T. mongolica* genome.

**Figure S6.** The KEGG pathway analysis of contracted genes in *T. mongolica* genome.

**Figure S7.** Distribution of *Copia* and *Gypsy* insertion events in different species.

**Figure S8.** Chromosome location and distribution analysis of *TmTPSs*. Tandem duplicated genes are linked by a red line.

dsad004_suppl_Supplementary_Figure

dsad004_suppl_Supplementary_Table

## Data Availability

All raw sequence data have been deposited at the NCBI under the BioProject PRJNA868131. The genome assembly, annotations, predicted CDS, and protein sequences are available on FigShare at the link: https://doi.org/10.6084/m9.figshare.20463798.v1.
