# Supplementary material for: Chromosome-level genome assembly of the endangered plant Tetraena mongolica
Source: DNA Res. 2023 Mar 31;30(2):dsad004. doi: 10.1093/dnares/dsad004 (PMC10113878; doi:10.1093/dnares/dsad004)
Supplement: dsad004_suppl_Supplementary_Figure [file dsad004_suppl_supplementary_figure.docx]

**Supplementary Figure**


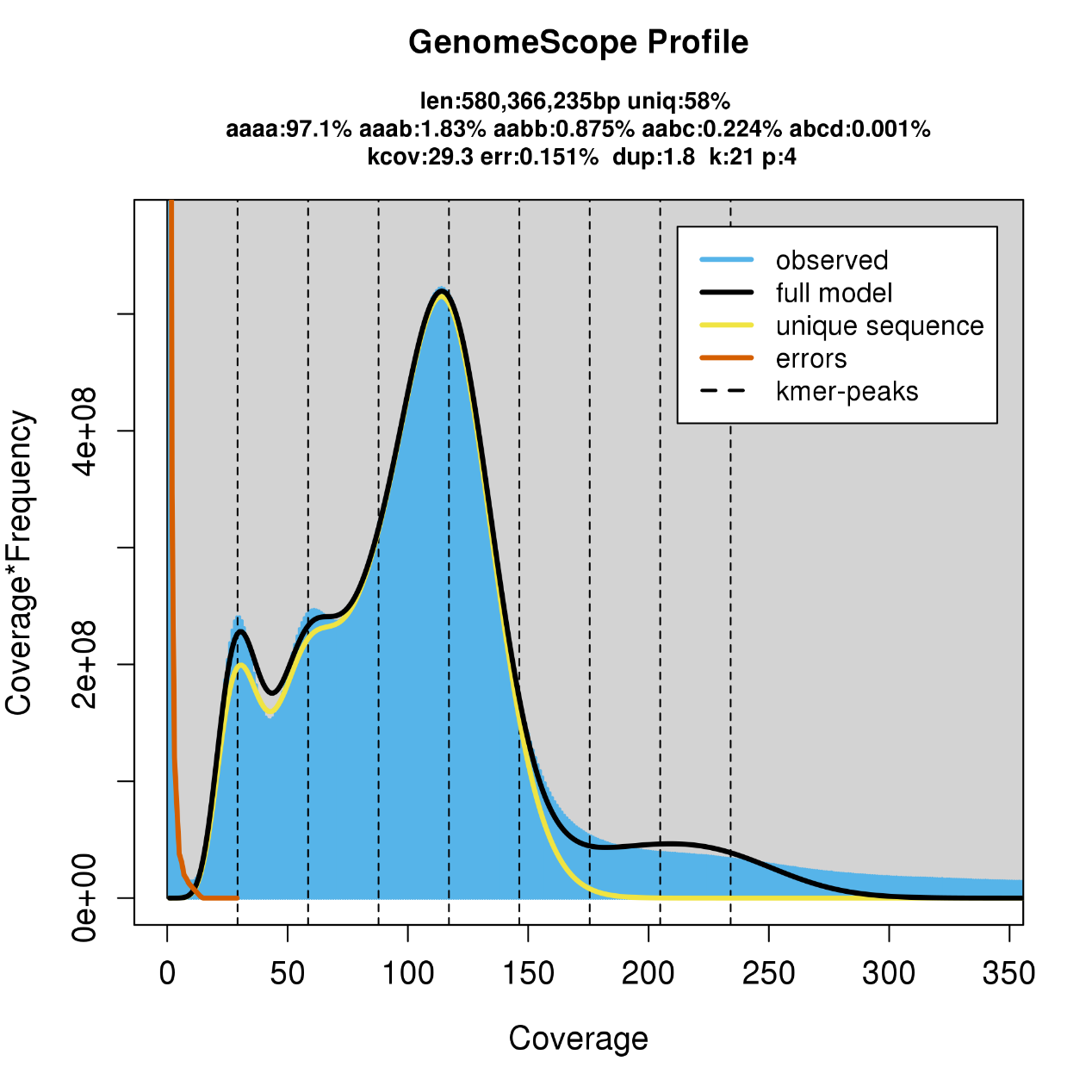


**Figure S1.** 21-mer analysis to estimate the *T. mongolica* genome size.


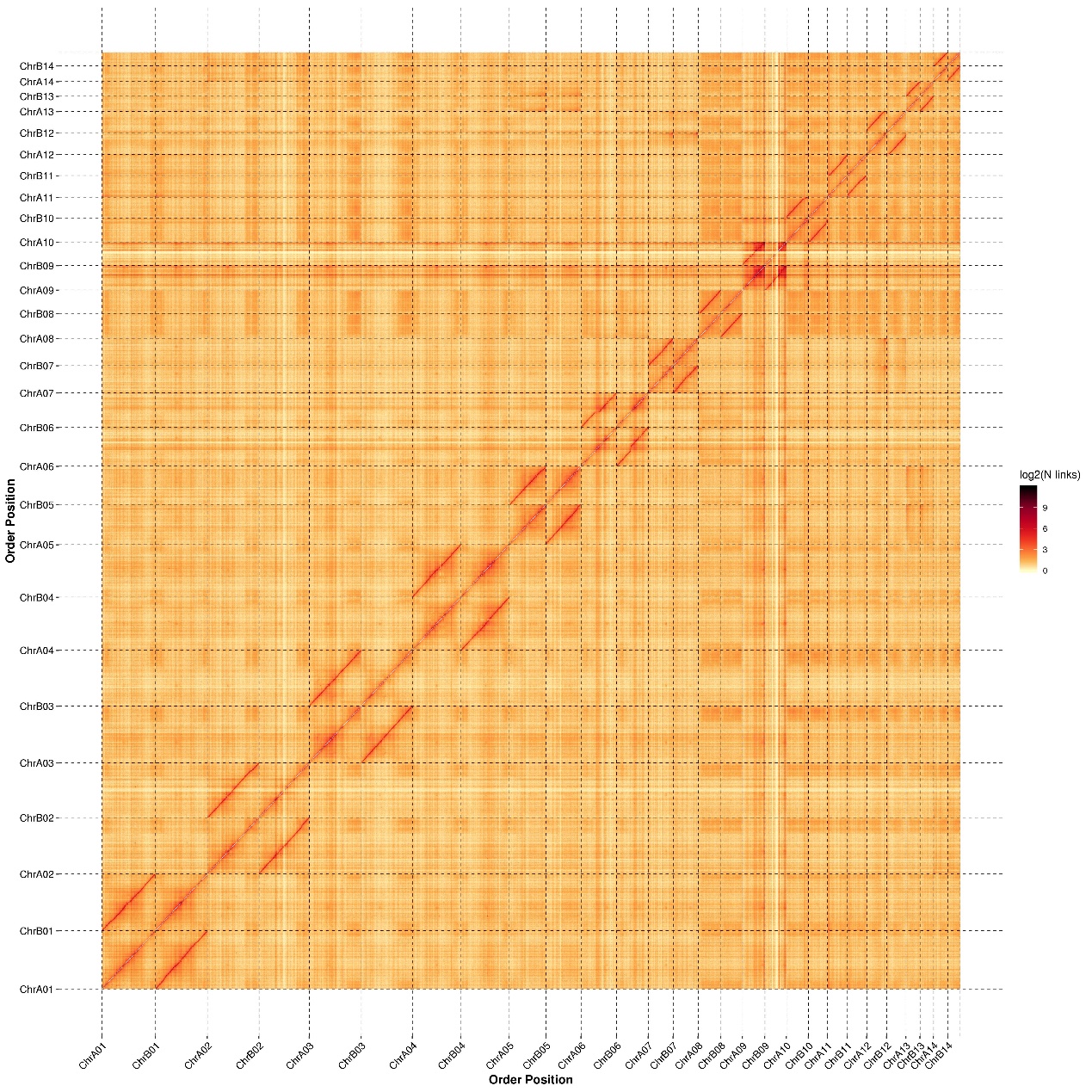


**Figure S2.** Heatmap of the Hi-C interaction density between 28 pseudochromosomes.


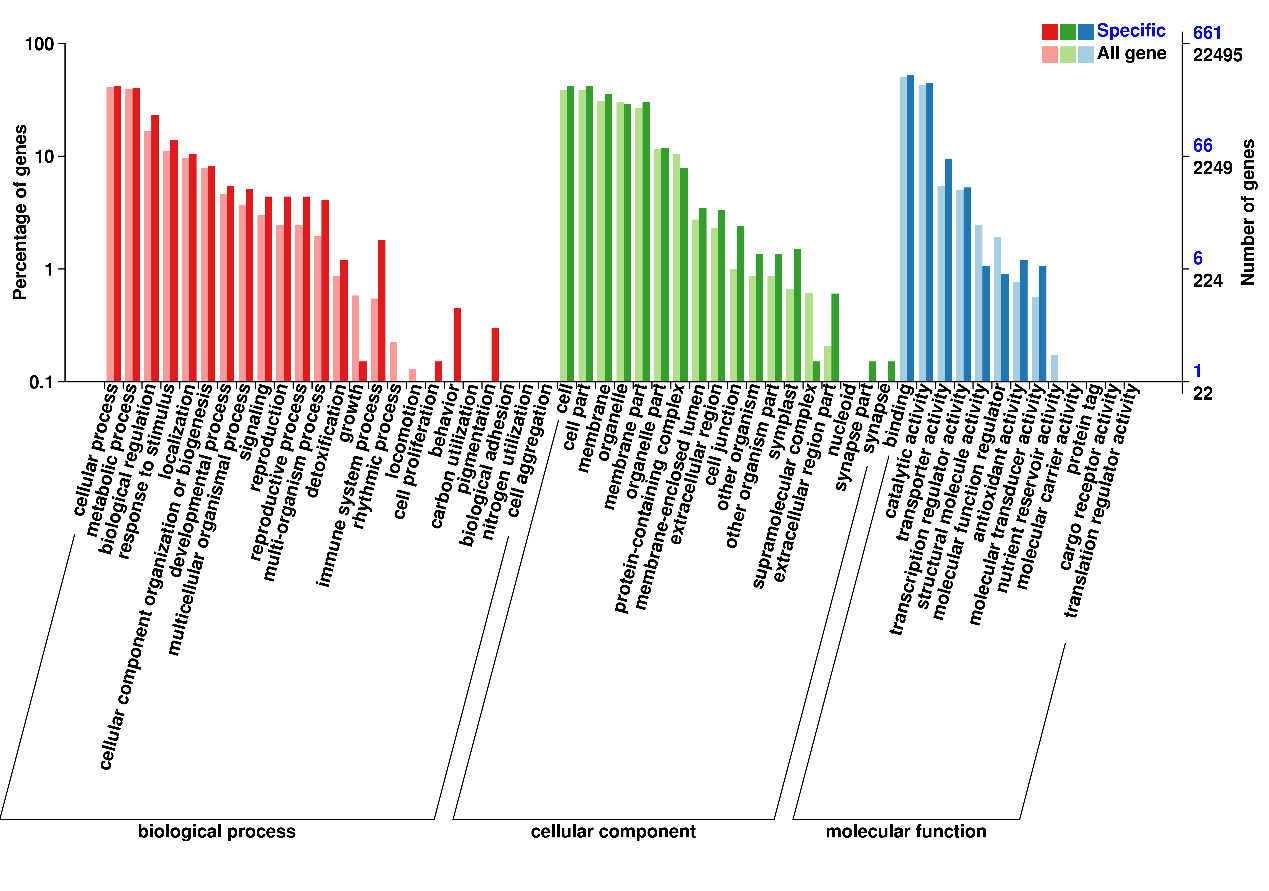


**Figure S3.** GO annotations of unique genes in *T. mongolica* genome.


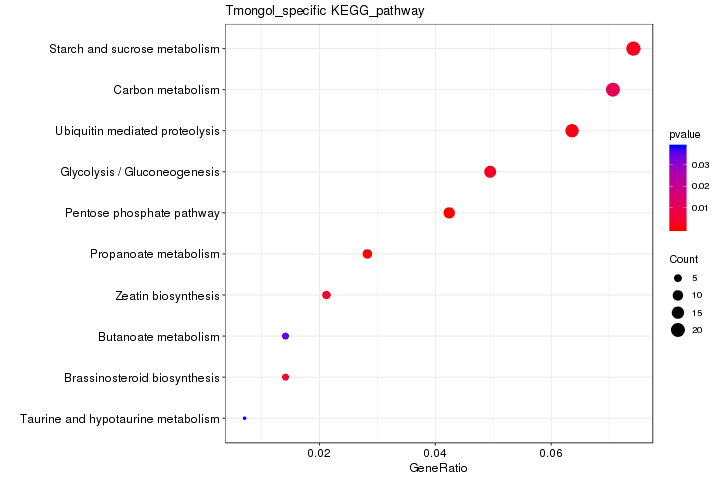


**Figure S4.** The KEGG pathway analysis of unique genes in *T. mongolica* genome.


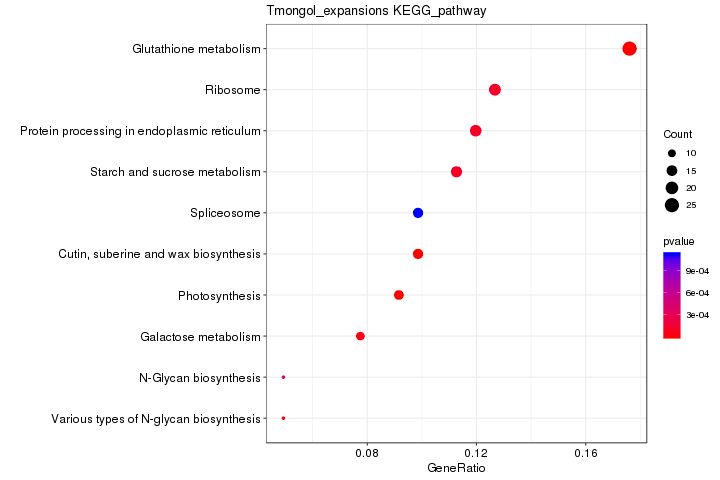


**Figure S5.** The KEGG pathway analysis of expanded genes in *T. mongolica* genome.


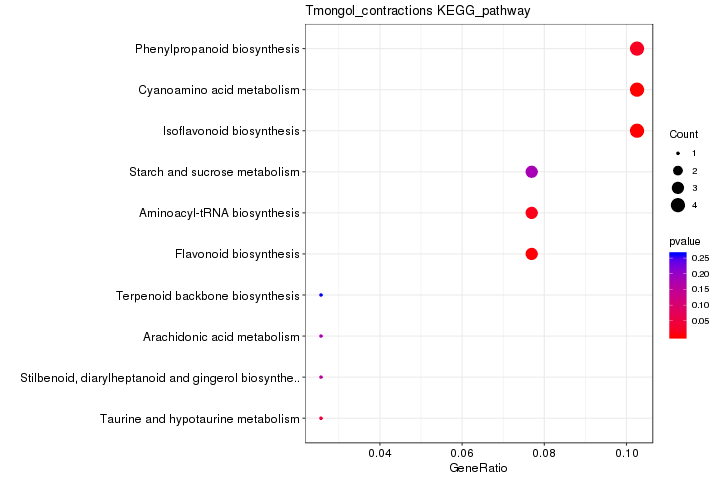


**Figure S6.** The KEGG pathway analysis of contracted genes in *T. mongolica* genome.


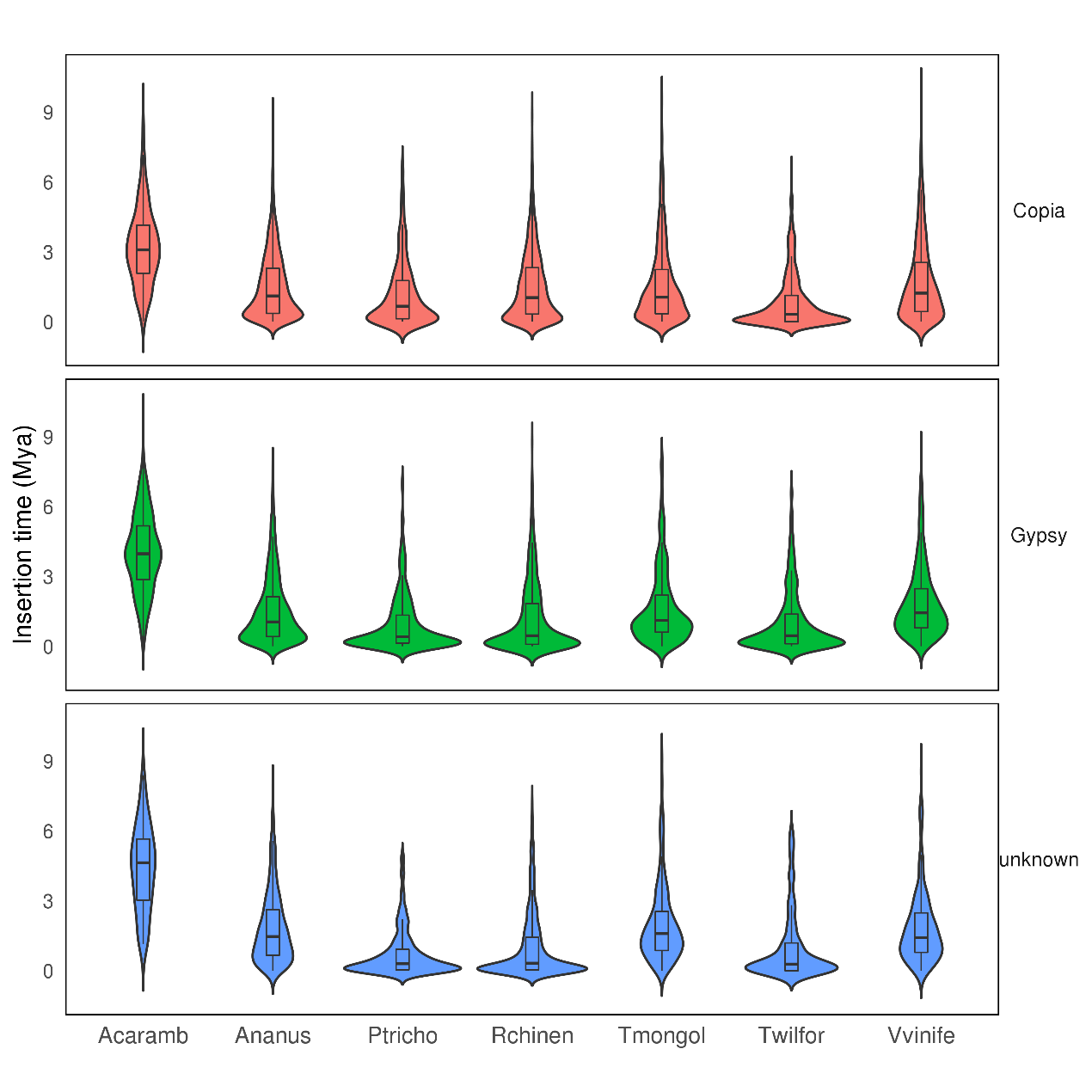


**Figure S7.** Distribution of *Copia* and *Gypsy* insertion events in different species.


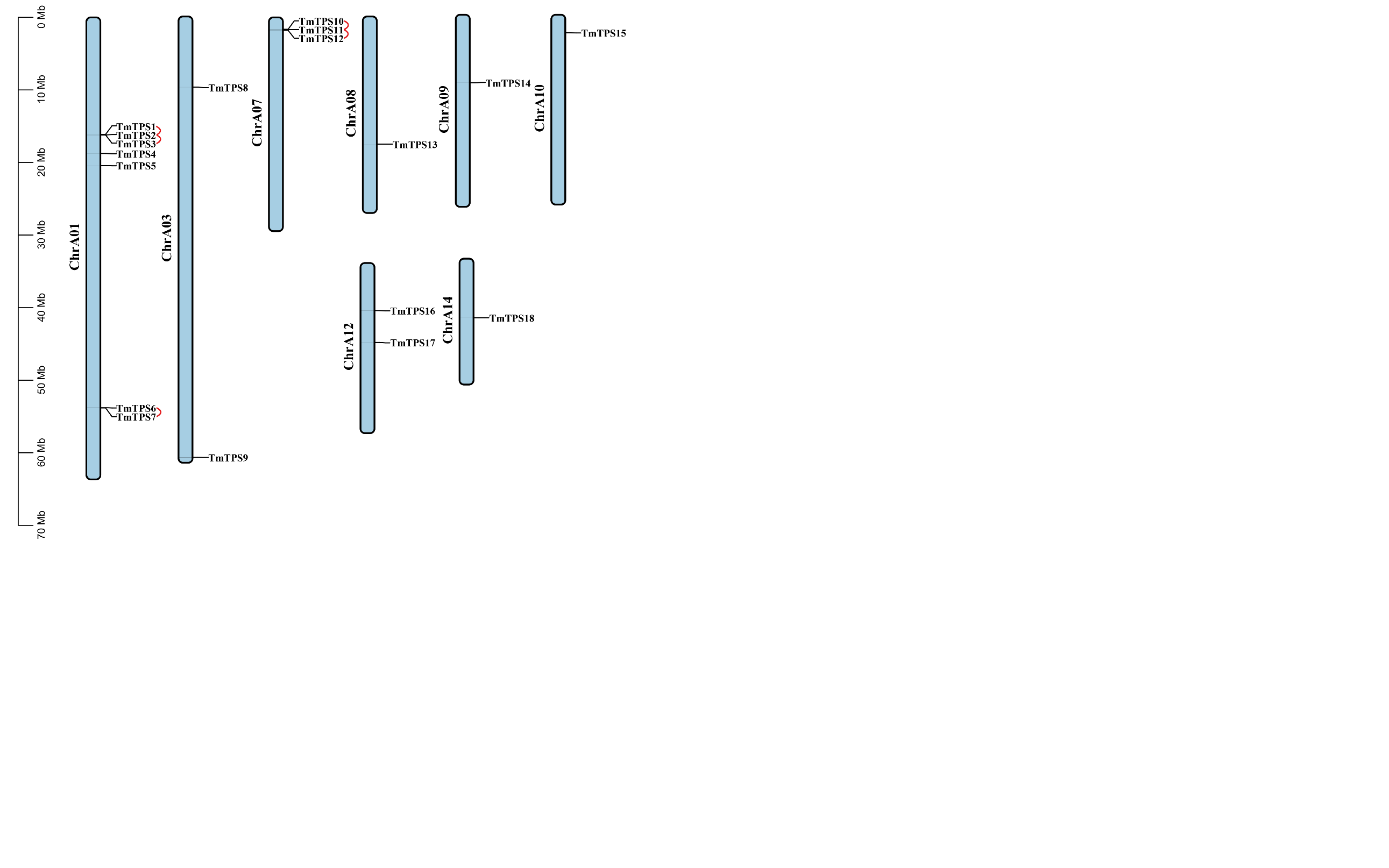


**Figure S8.** Chromosome location and distribution analysis of *TmTPSs*. Tandem duplicated genes are linked by a red line.
