# Supplementary material for: Chromosome-level genome assembly of the endangered plant Tetraena mongolica
Source: DNA Res. 2023 Mar 31;30(2):dsad004. doi: 10.1093/dnares/dsad004 (PMC10113878; doi:10.1093/dnares/dsad004)
Supplement: dsad004_suppl_Supplementary_Table [file dsad004_suppl_supplementary_table.docx]

**Supplementary Table**

|  | **Table S1.** Summary of sequencing data for *T. mongolica* | | | | |
| --- | --- | --- | --- | --- | --- |
| **Library type** | **Sequencing platform** | **Clean base** | **Clean reads** | **Read length** | **Application** |
| Long reads (CCS) | PacBio Sequel | 65.31 Gb | 4,179,497 | 15,995 bp (N50) | Genome assembly |
| Hi-C | Illumine HiSeq X Ten | 247.65 Gb | 826,151,353 | 150_150 bp | Chromosome construction |
| Short reads | Illumina HiSeq 2500 | 81.89 Gb | 265,357,126 | 150_150 bp | Genome estimation and polishing |
| Iso-seq | Pacbio Sequel II | 41.22 Gb | 47,426,327 | 1,626 bp (N50) | Genome annotation |

| **Table S2.** Estimation of genome size based on 21-mer statistics | | | |
| --- | --- | --- | --- |
| **Monoploid genome Size (Mb)** | **Diploid genome Size (Mb)** | **Heterozygosity rate (%)** | **GC content (%)** |
| 580.37 | 1,160.74 | 2.93 | 33.14 |

| **Table S3.** Statistics data of Hi-C assembly | | | | | |
| --- | --- | --- | --- | --- | --- |
| **Pseudochromosome** | **Cluster number** | **Length** | **Order number** | **length** |  |
| ChrA01 | 15 | 64,231,126 | 10 | 63,841,079 | |
| ChrB01 | 5 | 62,992,097 | 1 | 62,000,000 | |
| ChrA02 | 3 | 61,306,869 | 3 | 61,306,869 | |
| ChrB02 | 2 | 59,591,357 | 2 | 59,591,357 | |
| ChrA03 | 11 | 62,357,568 | 5 | 61,664,372 | |
| ChrB03 | 1 | 60,757,837 | 1 | 60,757,837 | |
| ChrA04 | 1 | 57,554,339 | 1 | 57,554,339 | |
| ChrB04 | 8 | 57,862,286 | 5 | 57,455,220 | |
| ChrA05 | 1 | 44,000,000 | 1 | 44,000,000 | |
| ChrB05 | 3 | 41,758,275 | 1 | 41,615,958 | |
| ChrA06 | 5 | 42,416,053 | 4 | 42,377,499 | |
| ChrB06 | 8 | 38,087,811 | 2 | 37,859,544 | |
| ChrA07 | 8 | 29,792,899 | 5 | 29,597,554 | |
| ChrB07 | 6 | 29,774,692 | 4 | 29,594,654 | |
| ChrA08 | 5 | 30,122,438 | 1 | 27,285,005 | |
| ChrB08 | 3 | 25,623,700 | 1 | 25,548,595 | |
| ChrA09 | 38 | 33,924,361 | 3 | 26,719,795 | |
| ChrB09 | 10 | 25,677,520 | 8 | 25,594,264 | |
| ChrA10 | 5 | 27,164,635 | 1 | 26,279,091 | |
| ChrB10 | 6 | 23,053,052 | 4 | 22,565,349 | |
| ChrA11 | 6 | 24,122,926 | 3 | 23,867,899 | |
| ChrB11 | 6 | 23,776,855 | 3 | 23,267,834 | |
| ChrA12 | 1 | 23,584,934 | 1 | 23,584,934 | |
| ChrB12 | 27 | 26,315,213 | 3 | 23,434,673 | |
| ChrA13 | 4 | 16,777,802 | 2 | 16,599,897 | |
| ChrB13 | 3 | 16,157,759 | 1 | 16,026,538 | |
| ChrA14 | 20 | 18,532,730 | 2 | 17,480,896 | |
| ChrB14 | 4 | 15,453,374 | 1 | 15,000,000 | |
| Total  (Ratio %) | 215  (25.75) | 1,042,770,508 (92.73) | 79  (36.74) | 1,022,471,052 (98.05) | |

| **Table S4.** Evaluation of completeness of the final genome assembly and annotation using BUSCO | | | | |
| --- | --- | --- | --- | --- |
| **Type** | **Assemmbly** | | **Annotation** | |
|  | **Number** | **Percent (%)** | **Number** | **Percent (%)** |
| Complete BUSCOs (C) | 1,533 | 94.98 | 1,587 | 98.33 |
| Complete and single-copy BUSCOs (S) | 299 | 18.53 | 132 | 8.18 |
| Complete and duplicated BUSCOs (D) | 1234 | 76.46 | 1455 | 90.15 |
| Fragmented BUSCOs (F) | 9 | 0.56 | 8 | 0.50 |
| Missing BUSCOs (M) | 72 | 4.46 | 19 | 1.18 |
| Total BUSCO groups searched | 1,614 | - | 1,614 | - |

| **Table S5.** Repeat annotations of the *T. mongolica* genome assembly | | | | |
| --- | --- | --- | --- | --- |
| **Order** | **Super family** | **Number of elements** | **Length of sequence (bp)** | **Percentage of sequence (%)** |
| SINE |  | 6,435 | 1,073,612 | 0.10 |
| LINE |  | 64,020 | 36,163,557 | 3.22 |
| DIRS |  | 5 | 304 | 0 |
| LTR |  | 401,465 | 325,271,909 | 28.93 |
|  | Copia | 111,244 | 119,521,156 | 10.63 |
|  | Gypsy | 98,873 | 114,740,918 | 10.20 |
|  | Caulimovirus | 2,668 | 4,014,248 | 0.36 |
|  | ERV | 3,892 | 295,081 | 0.03 |
|  | Ngaro | 1,231 | 97,896 | 0.01 |
|  | Pao | 1,497 | 1,212,405 | 0.11 |
|  | Unknow | 182,060 | 85,390,205 | 7.59 |
| DNA transposon |  | 383,672 | 140,884,468 | 12.53 |
|  | CACTA | 14,576 | 9,502,336 | 0.84 |
|  | Dada | 1,178 | 63,210 | 0.01 |
|  | Helitron | 1,260 | 264,940 | 0.02 |
|  | IS3EU | 768 | 62,224 | 0.01 |
|  | Kolobok | 2,155 | 274,322 | 0.02 |
|  | Merlin | 1,026 | 118,189 | 0.01 |
|  | Mutator | 23,343 | 17,646,681 | 1.57 |
|  | PIF-Harbinger | 3,761 | 414,165 | 0.04 |
|  | Tc1-Mariner | 704 | 118,278 | 0.01 |
|  | hAT | 21,158 | 16,249,755 | 1.44 |
|  | Unknown | 311,841 | 96,062,243 | 8.54 |
| Unknown |  | 133 | 10,208 | 0 |
| Unspecified |  | 1 | 36 | 0 |
| Total |  | 855,731 | 503,404,094 | 44.76 |
| Note: SINE, short interspersed element; LINE, long interspersed nuclear element; LTR, long terminal repeat. | | | | |

| **Table S6.** Comparison of the gene set of *T. mongolica* with other species | | | | | | | |
| --- | --- | --- | --- | --- | --- | --- | --- |
| **Species** | **Gene number** | **Average gene length (bp)** | **Average CDS length (bp)** | **Average exon length (bp)** | **Average intron length (bp)** | **Average exon number per gene** | **Average intro number per gene** |
| *T. mongolica* | 61,888 | 3,821.5 | 1,320.8 | 1,606.9 | 2,214.6 | 5.37 | 4.37 |
| *V. vinifera* | 26,346 | 6,454.0 | 1,137.1 | 1,137.1 | 5,316.9 | 5.95 | 4.95 |
| *A. thaliana* | 27,336 | 2,204.4 | 1,219.2 | 1,481.4 | 7,23.0 | 5.31 | 4.31 |
| *P. trichocarpa* | 41,335 | 3,108.4 | 1,158.8 | 1,158.8 | 1,949.6 | 4.79 | 3.79 |
| *M. truncatula* | 50,444 | 2,621.0 | 988.2 | 1,262.3 | 1,358.8 | 4.06 | 3.06 |

| **Table S7.** Functional annotation of the predicted genes for *T. mongolica* | | | |
| --- | --- | --- | --- |
| **Type** | | **Number** | **Percent (%)** |
| Annotation | NR | 58,581 | 94.66 |
|  | eggNOG | 51,129 | 82.62 |
|  | Pfam | 52,814 | 85.34 |
|  | Swissprot | 50,445 | 81.51 |
|  | TrEMNL | 58,922 | 95.21 |
|  | KOG | 34,561 | 55.84 |
|  | GO | 50,859 | 82.18 |
|  | KEGG | 45,318 | 73.23 |
| Total | Annotated | 59,761 | 96.56 |
|  | Gene | 61,888 | - |

| **Table S8.** Statistics of gene families in 12 plant species | | | | | | |
| --- | --- | --- | --- | --- | --- | --- |
| **Species** | **Total genes** | **Genes in families** | **Unclassified genes** | **Total families** | **Unique families** | **Genes in unique families** |
| *T. mongolica* | 27,481 | 24,476 | 3,005 | 13,707 | 348 | 973 |
| *T. wilfordii* | 31,295 | 29,845 | 1,450 | 14,415 | 387 | 1,637 |
| *V. vinifera* | 24,939 | 23,706 | 1,233 | 14,287 | 289 | 1,308 |
| *A. carambola* | 24,531 | 21,581 | 2,950 | 14,238 | 306 | 1,049 |
| *A. nanus* | 34,886 | 30,886 | 4,000 | 15,525 | 695 | 2,969 |
| *A. thaliana* | 27,316 | 24,026 | 3,290 | 13,532 | 734 | 3,189 |
| *A. trichopoda* | 16,599 | 15,358 | 1,241 | 12,257 | 192 | 696 |
| *H. ammodendron* | 41,533 | 32,201 | 9,332 | 14,910 | 2,514 | 13,536 |
| *M. truncatula* | 49,906 | 37,787 | 12,119 | 16,695 | 1,836 | 8,670 |
| *O. sativa* | 28,317 | 24,245 | 4,072 | 13,310 | 1,406 | 5,433 |
| *P. trichocarpa* | 34,584 | 31,222 | 3,362 | 15,002 | 411 | 1,421 |
| *R. chinensis* | 45,464 | 33,535 | 11,929 | 17,330 | 2,239 | 7,602 |

| **Table S9.** *TmTPSs* information for *T. mongolica* | | | | | | |
| --- | --- | --- | --- | --- | --- | --- |
| **Name** | **Gene ID** | **Chromosome location** | | | **Protein length** | **TPS subfamily** |
| *TmTPS1* | TmoA01G010990.1 | ChrA01 | 16110319 | 16116523 | 602 | a |
| *TmTPS2* | TmoA01G011010.1 | ChrA01 | 16198739 | 16204835 | 598 | a |
| *TmTPS3* | TmoA01G011020.1 | ChrA01 | 16211644 | 16219724 | 596 | a |
| *TmTPS4* | TmoA01G011970.1 | ChrA01 | 18757508 | 18761555 | 430 | a |
| *TmTPS5* | TmoA01G012620.1 | ChrA01 | 20419977 | 20423638 | 552 | a |
| *TmTPS6* | TmoA01G022550.1 | ChrA01 | 53792250 | 53806543 | 660 | e/f |
| *TmTPS7* | TmoA01G022560.1 | ChrA01 | 53816718 | 53823885 | 857 | e/f |
| *TmTPS8* | TmoA03G006020.1 | ChrA03 | 9727472 | 9749885 | 740 | e/f |
| *TmTPS9* | TmoA03G031840.1 | ChrA03 | 60751824 | 60760630 | 839 | c |
| *TmTPS10* | TmoA07G001050.1 | ChrA07 | 1642326 | 1648207 | 543 | a |
| *TmTPS11* | TmoA07G001060.1 | ChrA07 | 1682328 | 1686950 | 559 | a |
| *TmTPS12* | TmoA07G001070.1 | ChrA07 | 1787759 | 1792628 | 562 | a |
| *TmTPS13* | TmoA08G013280.1 | ChrA08 | 17595417 | 17598248 | 614 | a |
| *TmTPS14* | TmoA09G003490.1 | ChrA09 | 9362602 | 9366290 | 560 | g |
| *TmTPS15* | TmoA10G002060.1 | ChrA10 | 2485506 | 2489660 | 592 | a |
| *TmTPS16* | TmoA12G005560.1 | ChrA12 | 6553595 | 6557276 | 614 | b |
| *TmTPS17* | TmoA12G009090.1 | ChrA12 | 10939159 | 10945048 | 797 | e/f |
| *TmTPS18* | TmoA14G005470.1 | ChrA14 | 8139721 | 8143642 | 558 | b |

| **Table S10.** Information of genes involved in triacylglycerol biosynthesis in *T. mongolica* | | | | | |
| --- | --- | --- | --- | --- | --- |
| **Name** | **Gene ID** | **Chromosome location** | | | **Protein length** |
| TmGPAT1 | TmoA03G029930.1 | ChrA03 | 59058127 | 59060064 | 498 |
| TmGPAT2 | TmoA04G001130.1 | ChrA04 | 1179215 | 1181809 | 503 |
| TmGPAT3 | TmoA05G014500.1 | ChrA05 | 33570765 | 33572844 | 547 |
| TmGPAT4 | TmoA08G006280.1 | ChrA08 | 8294979 | 8297794 | 549 |
| TmGPAT5 | TmoA08G021460.1 | ChrA08 | 26535262 | 26537145 | 334 |
| TmGPAT6 | TmoA12G000950.1 | ChrA12 | 1120833 | 1125337 | 531 |
| TmGPAT7 | TmoA12G006820.1 | ChrA12 | 8001772 | 8004547 | 502 |
| TmGPAT8 | TmoA13G011480.1 | ChrA13 | 15508357 | 15511170 | 513 |
| TmGPAT9 | TmoA14G012620.1 | ChrA14 | 16709604 | 16712029 | 543 |
| TmLPAT1 | TmoA04G003630.1 | ChrA04 | 4166117 | 4170600 | 384 |
| TmLPAT2 | TmoA08G000760.1 | ChrA08 | 1245709 | 1249754 | 269 |
| TmLPAT3 | TmoA08G007530.1 | ChrA08 | 9971153 | 9974687 | 386 |
| TmLPAT4 | TmoA09G000650.1 | ChrA09 | 1170694 | 1176564 | 386 |
| TmLPAT5 | TmoA13G007210.1 | ChrA13 | 10309252 | 10317506 | 391 |
| TmLPAT6 | TmoA14G007690.1 | ChrA14 | 10848420 | 10851006 | 384 |
| TmLPEAT1 | TmoA03G002220.1 | ChrA03 | 3007959 | 3014945 | 378 |
| TmLPEAT2 | TmoA04G016560.1 | ChrA04 | 43353705 | 43360287 | 546 |
| TmLPEAT3 | TmoA08G020640.1 | ChrA08 | 25542301 | 25548615 | 404 |
| TmLPEAT4 | TmoA10G010810.1 | ChrA10 | 12606266 | 12613382 | 533 |
| TmLPEAT5 | TmoA11G016450.1 | ChrA11 | 21350655 | 21358683 | 385 |
| TmLPEAT6 | TmoA01G013480.1 | ChrA01 | 25094365 | 25099637 | 470 |
| TmPP1 | TmoA03G008850.1 | ChrA03 | 20168267 | 20170543 | 725 |
| TmPP2 | TmoA08G007990.1 | ChrA08 | 10468260 | 10475770 | 940 |
| TmPP3 | TmoA08G010600.1 | ChrA08 | 14388920 | 14395066 | 691 |
| TmPP4 | TmoA08G010620.1 | ChrA08 | 14424738 | 14427965 | 735 |
| TmPP5 | TmoA08G020160.1 | ChrA08 | 25071838 | 25079067 | 1150 |
| TmPP6 | TmoA10G009480.1 | ChrA10 | 11226939 | 11228776 | 767 |
| TmPP7 | TmoA12G006000.1 | ChrA12 | 6948872 | 6955048 | 930 |
| TmWSD1 | TmoA01G009570.1 | ChrA01 | 12863196 | 12870703 | 487 |
| TmWSD2 | TmoA01G009590.1 | ChrA01 | 12885722 | 12891905 | 322 |
| TmWSD3 | TmoA01G015680.1 | ChrA01 | 40832326 | 40837555 | 480 |
| TmWSD4 | TmoA01G029980.1 | ChrA01 | 63419637 | 63429877 | 254 |
| TmWSD5 | TmoA01G029990.1 | ChrA01 | 63437767 | 63442399 | 495 |
| TmWSD6 | TmoA04G007960.1 | ChrA04 | 9743449 | 9748632 | 492 |
| TmWSD7 | TmoA04G007970.1 | ChrA04 | 9759723 | 9762382 | 486 |
| TmWSD8 | TmoA08G017980.1 | ChrA08 | 22815869 | 22822657 | 490 |
| TmWSD9 | TmoA09G002240.1 | ChrA09 | 4852177 | 4864880 | 487 |
| TmWSD10 | TmoA10G011710.1 | ChrA10 | 13599078 | 13603754 | 534 |
| TmWSD11 | TmoA10G011720.1 | ChrA10 | 13611445 | 13616572 | 523 |
| TmDGAT1 | TmoA02G008950.1 | ChrA02 | 25478764 | 25487447 | 327 |
| TmDGAT2 | TmoA04G004930.1 | ChrA04 | 5821192 | 5823077 | 350 |
| TmDGAT3 | TmoA04G023070.1 | ChrA04 | 54909008 | 54917892 | 502 |
| TmDGAT4 | TmoA10G003670.1 | ChrA10 | 4513718 | 4522144 | 501 |
